# The Effect of Reciting the Quran on the Anxiety of Neurosurgery Muslim Candidates

**DOI:** 10.1002/hsr2.70629

**Published:** 2025-04-08

**Authors:** Nader Salari, Reza Fayzi, Elahe Abdipuor, Hooman Ghasemi, Shamarina Shohaimi, Masoud Mohammadi

**Affiliations:** ^1^ Department of Biostatistics, School of Health Kermanshah University of Medical Sciences Kermanshah Iran; ^2^ Department of Operating Room, School of Allied Medical Sciences University of Medical Sciences Arak Iran; ^3^ Student Research Committee Kermanshah University of Medical Sciences Kermanshah Iran; ^4^ Department of Biology, Faculty of Science University Putra Malaysia Serdang Malaysia; ^5^ Research Center for Social Determinants of Health Jahrom University of Medical Sciences Jahrom Iran

**Keywords:** anxiety, candidate for neurosurgery, quranic voice

## Abstract

**Background:**

Anxiety is very common among patients undergoing surgery. Like other forms of surgery, neurosurgery can affect patients both physically and emotionally. One of the ways to control and reduce anxiety is to pay attention to spiritual interventions and spiritual health. The present study aimed to investigate the effect of the Qur'an on the anxiety of neurosurgery Muslim candidates.

**Methods:**

This study is interventional research with a pretest and posttest design with a control group. The research population included all patients who were candidates for brain and nerve surgery in the teaching‐treatment hospitals in 2019. The samples were selected by probability and simple random sampling methods. The data collection tool included the Spielberger questionnaire and demographics (age, gender, marital status, education degree, job, etc.) and the vital signs checklist (blood pressure, heart rate, and respiration).

**Results:**

The results of this study showed that the sound of the Quran leads to a decrease in the level of anxiety in the experimental group. The effect size of the group for overt anxiety and anxiety subscales is 53.5% and 40%, respectively; blood pressure is 4.3%, breathing is 20.9%, and heart rate is 21.5%.

**Conclusion:**

Anxiety is an unpleasant complication that many people suffer from before various surgeries. The results of this study indicated that the sound of the Quran is a positive and effective factor in reducing anxiety before neurosurgery in Muslim patients who are candidates for this surgery, so health policymakers can use the results of this study as a research priority to reduce anxiety before surgery.

## Summary

- Anxiety is an unpleasant complication that many people suffer from before various surgeries.
- The results of this study showed that the sound of the Quran leads to a decrease in the level of anxiety in the experimental group.
- The results of this study indicated that the sound of the Quran is a positive and effective factor in reducing anxiety before neurosurgery in Muslim patients who are candidates for this surgery.

## 1 Introduction

Anxiety is very common among patients undergoing surgery [1]. Like other forms of surgery, neurosurgery can affect patients both physically and emotionally. For example, craniotomy has been observed to induce significant anxiety and fear in patients during surgery [2, 3], which can cancel or delay surgery [4]. Therefore, it is very important to control and overcome the stress and anxiety of surgery.

Various nonpharmacological methods such as exercise, diet, relaxation techniques, book therapy [5, 6, 7], and soothing music [8] are among the complementary and alternative medicine methods that are used to reduce sympathetic reactions [9]. Since these methods are effective, cheap, low‐risk, and noninvasive and do not require special time and expensive equipment, the treatment staff can easily implement them along with other nursing care [10].

Therapeutic music is an effective and safe intervention to reduce anxiety, reduce feelings of loneliness and excitability, improve mood, and facilitate emotions [11], which improves the mental, physical, emotional, and social health of people [12]. Therefore, music may be an effective adjunct to antianxiety medications such as midazolam, with the added benefit of potentially reducing indicators of intraoperative stress, including heart rate and blood pressure [13, 14]. Also, many nonpharmacological methods, such as spiritual power, are effective in managing preoperative anxiety [1].

The soul‐enhancing music of reciting the Holy Quran is one of the most beautiful natural music [11]. Reciting the Quran and listening to the recitation of the Quran is one of the nonpharmacological approaches, such as psychological methods and cognitive therapies, that are used to manage anxiety [15].

Based on a study conducted in the field of reviewing the effect of reciting and listening to the Holy Quran on anxiety, stress, and depression, it has been reported that reciting and listening to the Quran can be used as a useful nonpharmacological treatment for reducing anxiety, stress, and depression [16]. According to another review in this field, it is reported that based on the available research, reciting the Quran can be used as a useful nonpharmacological treatment for reducing anxiety [17].

Qur'an is a divine book with the purpose of the spiritual guidance of man [18] and contains important advice for human happiness in worldly life and life after death [19]. In addition, many verses contain instructions for achieving mental health and reducing human suffering and stress, as the Holy Prophet, may God bless him and grant him peace, said: Be aware that the remembrance of God calms the hearts [20].

Considering the high prevalence of anxiety in neurosurgery candidates and the importance of complementary and alternative medicine methods, we tried to do a general statistical study on the effect of reciting the Quran on the anxiety of neurosurgery candidates through scientific research. The purpose of this study is to investigate the effect of the sound of the Quran on the anxiety of Muslim patients who are candidates for neurosurgery, which can be important evidence for considering complementary and alternative medicine methods, including the effect of the sound of the Quran, to reduce anxiety in these patients.

## 2 Methods

This study is interventional research with a pretest and posttest design with a control group. The research population included all Muslim patients who were candidates for neurosurgery in the teaching‐treatment hospitals from the beginning of the year until the end of 2020.

The studied samples were selected from patients who were interested in participating in the research and had the characteristics determined by the physician. The sample size in this study was determined according to the results and research method in similar studies, including the research of Aghajani et al. [21], and the formula for determining the sample was $n=\frac{{{\left(Z_{1-\frac{\propto }{2}}+Z_{1-\beta }\right)}^{2}*\sigma }^{2}}{{\left(\mu_{1}-\mu_{2}\right)}^{2}}\;$ and the number of patients for neurosurgery in educational hospitals was estimated to be 60 people, 30 people in the test group and 30 people in the experimental group randomly. Witnesses were placed on a daily basis.

The research units were hospitalized for the first time for brain and nerve auction; they did not have hearing problems, addictions, or mental diseases. Also, before the participation of the patients in the study, the level of anxiety was assessed using the Spielberger Anxiety Scale, and patients who scored 41 or higher on this questionnaire and who did not use painkillers and anti‐anxiety drugs were included in the study. The age range was 20–60 years, and written informed consent was among the other inclusion criteria that were considered for this study.

The data collection tools in this study included a personal information questionnaire, Spielberger anxiety scale, and vital sign registration checklist. The Spielberger Anxiety Scale was first published in 1973. This test has two scales: situational anxiety and personality anxiety. Each of these scales has 20 statements designed as a 4‐point Likert scale (not at all, sometimes, often, and always) and has a weight between 1 and 4. Therefore, the scores of each of the two scales vary between 20 and 80. Based on that, people who have mild anxiety (score 20–40), moderate anxiety (score 41–60), and severe anxiety (score 61–80). The version of the Spielberger anxiety scale used in this study was standardized by Mehram in Mashhad. He obtained the validity of this scale for overt anxiety by calculating Cronbach's *α* coefficient of 0.91, for the covert anxiety scale of 0.90, and for the whole test 0.94 [22]. A mercury sphygmomanometer and a digital wristwatch were used to measure vital signs. The participants were placed under the same conditions and were controlled in terms of taking analgesic medications and engaging in calming conversations.

The control group samples receive a standard protocol that is intended for all patients to create psychological preparation and control anxiety before neurosurgery. The general principles of this protocol include informing, educating, and familiarizing the patient with the surgery and its complications by avoiding the use of specialized terms. The necessary awareness was provided to patients and even their relatives and companions by providing them with necessary pamphlets and brochures before the operation, and verbal education was also provided through face‐to‐face meetings and encouragement, reducing anxiety, and preparing the patient for the surgical team before the operation at the patient's bedside. 

The Intervention group samples were taken to a secluded room before entering the operating room. They listened to selected verses from the Qur'an (Surah Fajr and Hijrat) for 20 min using headphones, in the voice of Mohammad Sadegh Maneshavi. In the end, anxiety and vital signs were measured again.

Data analysis was done using SPSS statistical software version 24 at a confidence level of 95%. Quantitative variables were reported as mean ± standard deviation, and qualitative variables were reported as percentages. Analysis of covariance was used to analyze the hypotheses of the research. A *p* value of less than 0.05 was considered significant.

## 3 Results

In this study, 60 patients participated in two experimental and control groups. The mean and standard deviation of age in the experimental group was 44.5 ± 9.5 years and in the control group was 45.2 ± 9.9 years. The results of the Kolmogorov–Smirnov test showed that the age distribution was normal (*p* > 0.05). The results of the independent *t*‐test showed that there was no significant difference in age between the two groups (*p* = 0.782). The results of the *χ* ^2^ test showed that there was no statistically significant difference between gender (*p* = 0.371), marital status (*p* = 0.266), education (*p* = 0.147), occupation (*p* = 0.500), and history of blood pressure (*p* = 0.761). The results are presented in Table 1.

**Table 1:** Demographic variables of the examined samples in the study.

Variables	Groups	Control	Test	*p* value
		*N* (%)	*N* (%)	
Age	(*χ* ± SD)	44.5 ± 9.5	45.3 ± 9.9	0.782
Sex	Female	21 (70)	24 (80)	0.371
	Male	9 (30)	6 (20)	
Education	Below diploma	15 (50)	20 (66.7)	0.147
	Diploma (completion of education before entering the university)	15 (50)	10 (33.3)	
Marital status	Married	8 (26.7)	5 (16.7)	0.266
	Single	22 (73.3)	25 (83.3)	
Occupation	Homemaker (a woman who does not have a job and stays at home)	22 (73.3)	21 (70)	0.500
	Other	8 (26.7)	9 (30)	
History of blood pressure	No	22 (73.3)	24 (80)	0.761
	Yes	8 (26.7)	6 (20)	

Descriptive indices (mean and standard deviation) of overt anxiety and covert anxiety scores, blood pressure, heart rate, and breathing in the experimental and control groups in the pretest and posttest phases are presented in Table 2. The results of the independent *t*‐test showed that there was no statistically significant difference between the test and control groups in the pretest (*p* > 0.05). Also, the results of the paired *t*‐test showed that the sound of the Quran had a significant effect on reducing the scores of overt anxiety, covert anxiety, blood pressure, heart rate, and breathing (*p* < 0.05).

**Table 2:** Descriptive of state anxiety and vein anxiety scores.

Variable	Groups/Time	Before	After	*p* value**
		*χ* ± SD	*χ* ± SD	
Overt anxiety	Test	55.6 ± 12.5	38.5 ± 9.4	0.001
	Control	56.4 ± 11.7	56.03 ± 12.8	0.488
*p* value***				
Covert anxiety	Test	57.1 ± 12.4	43.4 ± 12.5	0.001
	Control	55.4 ± 11.1	58.2 ± 11.4	0.122
*p* value				
Anxiety (total)	Test	112.7 ± 24.9	81.9 ± 19.8	0.001
	Control	111.8 ± 23.7	113.3 ± 25.1	0.545
*p* value***				
Blood pressure	Test	137.3 ± 22.07	127.03 ± 18.4	0.001
	Control	133.8 ± 32.1	129.5 ± 30.7	0.096
*p* value***		0.623	0.706	
Pulse	Test	96.9 ± 10.4	88.9 ± 11.4	0.001
	Control	94.9 ± 10.6	93.03 ± 10.9	0.062
*p* value***		0.459	0.158	
Breathing	Test	18.5 ± 3.2	14.9 ± 3.1	0.001
	Control	17.7 ± 3.2	16.8 ± 3.1	0.045
*p* value***		0.363	0.025	

*p value < 0.05

** Paired *t*‐test.

*** Independent *t*‐test.

Before performing covariance analysis, the Shapiro–Wilk and Levene's test was used to check compliance with the necessary assumptions. The Shapiro–Wilk test for the distribution of the research variables in the posttest stage showed that the research variables have a normal distribution (*p* > 0.05). Lune's test was used to check the assumption of homogeneity of error variances. The results of Lune's test indicated that the assumption of homogeneity of variances is not rejected (*p* > 0.05). Examining the homogeneity of the regression slopes also showed that the assumption of the homogeneity of the regression slopes is maintained (*p* > 0.05). Therefore, there were prerequisites for performing multivariate covariance analysis. In Table 3, descriptive indices are mentioned along with the results of covariance analysis.

**Table 3:** The results of multivariate covariance analysis to investigate the effect of the Qur'an on the anxiety of neurosurgery candidates.

Source of variation	Dependent variable		Sum of squares	Degrees of freedom	Mean of squares	*F*	*p* value	Square of *η*
Group	Anxiety	Overt anxiety	4190.7	1	4190.7	64.3	0.001	0.535
		Covert anxiety	2958.8	1	2958.8	37.3	0.001	0.400
Group	Blood pressure		458.4	1	458.4	2.5	0.120	0.043
	Breathing		83.4	1	83.4	14.5	0.001	0.209
	Heart rate		546.2	1	546.2	15.1	0.001	0.215

The results of the multivariate covariance analysis in Table 3 show that there is a statistically significant difference between the experimental and control groups in the subscales of overt anxiety and covert anxiety in the posttest after removing the effect of the pretest (*p* < 0.05). In short, it can be said that the sound of the Quran leads to a decrease in the level of anxiety in the experimental group. The effect size of the group for the subscales of overt anxiety and covert anxiety is 53.5% and 40%, respectively; blood pressure is 4.3%, respiration is 20.9%, and heart rate is 21.5%.

## 4 Discussion

From a neurocognitive point of view, music is a sound language and a complex structure that stimulates the human brain at the sensory, motor, perceptual‐cognitive, and emotional levels at the same time and stimulates and integrates the neural pathways in a specific way [23]. Based on this study results, the sound of the Quran leads to a decrease in the level of anxiety in the experimental group. The effect size of the group for overt anxiety and anxiety subscales is 53.5% and 40%, respectively; blood pressure is 4.3%, breathing is 20.9%, and heart rate is 21.5%. The results of this study indicated that the sound of the Quran is a positive and effective factor in reducing anxiety before neurosurgery in Muslim patients who are candidates for this surgery.

There is published evidence of intraoperative musical intervention in 1809 when Jane Todd Crawford played hymns during the first oophorectomy without the use of anesthesia [24]. Since then, the possible benefits of intraoperative music intervention have been widely investigated, and increasing evidence shows its effectiveness in reducing intraoperative anxiety, pain, and stress in patients [13, 14, 25, 26, 27]. The results of another study, along with other studies, have reported that the use of music can reduce the pain level of patients after surgery [28, 29, 30].

The miracle hidden in the music and phonetic order of the Qur'an is one of the dimensions of the miracle of the Qur'an, which lies in the depth of the verses. The music of the Quran is a kind of miracle of expression. The verses of the Qur'an put all people under the influence of their words, even if the audience does not know the verses [31, 32, 33]. Pargament et al. (2014) believed that spirituality and religion can be effective in patients by creating a feeling of support and motivation [34]. Also, religious spirituality as a source of support facilitates coping with stress [35, 36, 37, 38, 39, 40].

Some mental disorders, such as depression and anxiety, are caused by damage to a person's spirituality, and when these damages are removed, mental problems are also cured [41, 42, 43]. The results of another study have reported that patients with spiritual inclinations are able to find meaning and purpose in their lives despite the crisis of the disease, and they are more successful in dealing with the disease and getting out of the crisis [44]. On the other hand, the results of several studies have shown that Quran recitation is more effective in reducing anxiety than other types of music [45, 46, 47].

Muslim patients generally believe in the healing power of the Quran. A cross‐sectional multistage cluster survey was conducted among 1408 people from the Saudi Arabian household. The results showed that 68% of the respondents have used recitation of the Holy Quran as a method for healing and treatment in the last 12 months [48]. Also, theologians, doctors, and researchers have strongly encouraged the use of religion‐based treatments, such as listening to the Holy Quran, as a way to strengthen traditional treatments in Muslim countries [49, 50, 51, 52].

Ansari‐Jabri et al. (2005) were one of the first to report that listening to recitations from the Holy Quran may be beneficial in reducing depression, especially in those who have a strong belief in the Holy Qur'an. These findings suggest that reciting the Holy Quran may be used as a nonpharmacological treatment to complement existing treatments in the treatment of dialysis patients suffering from depression [52]. The results of a study showed that the sound of the Quran is effective in reducing anxiety in women before cesarean section [53].

On the one hand, the cost of hospitalization in the ICU is high, and on the other hand, the mortality rate is high. Therefore, there is a need for effective management of these patients. In this regard, studies have been conducted on nonpharmacological interventions, such as listening to music, and spiritual interventions, such as prayer, and the results of these studies showed positive effects in reducing stress and anxiety in mechanically ventilated patients [54, 55, 56, 57, 58, 59, 60, 61, 62, 63, 64, 65, 66, 67, 68].

According to the study of al‐Shahat et al. the level of anxiety before surgery is different between minor and major surgeries, and it is higher that they recommend psychological counseling in hospitals to help patients reduce anxiety, especially for major surgeries they do. The results of their study showed that reciting the Quran has a significant effect in reducing anxiety before major and minor elective surgeries [69, 70, 71, 72].

One of the major limitations of the current study is that the current research is specific to neurosurgery and only in Muslim people, and it is recommended to conduct other research for other types of surgery and also to know the effect of the sound of the Quran on non‐Muslims. Muslims should also conduct research on this population.

## 5 Conclusion

Anxiety before surgery, especially major surgery such as neurosurgery, is abundant in Muslim patients who are candidates for surgery. Therefore, it is necessary to identify and use methods that are simple and cost‐effective in addition to reducing anxiety before surgery. According to the results of the present study, the sound of the Quran is one of the useful and effective methods in reducing the anxiety of patients before neurosurgery.

## Author Contributions

**Nader Salari:** conceptualization, software. **Reza Fayzi:** investigation, writing – original draft, writing – review and editing. **Elahe Abdipuor:** writing – original draft, resources, supervision. **Hooman Ghasemi:** supervision, writing – original draft, writing – review and editing. **Shamarina Shohaimi:** writing – review and editing, writing – original draft. **Masoud Mohammadi:** conceptualization, investigation, methodology, writing – review and editing, writing – original draft, supervision.

## Ethics Statement

This study was presented to the Research Council of Kermanshah University of Medical Sciences and was approved before conducting the study with the code of ethics under the number IR.KUMS.REC.1399.824 (Approval Date: 2020‐11‐21). All procedures performed in this study were performed in accordance with the ethical standards contained in the Declaration of Helsinki and its subsequent amendments or comparable ethical standards.

## Consent

Informed consent was obtained from all participants in the study.

## Conflicts of Interest

The authors declare no conflicts of interest.

### 1 Transparency Statement

The authors affirm that this manuscript is an honest, accurate, and transparent account of the study being reported.

## Data Availability

The data that support the findings of this study are available from the corresponding author upon reasonable request. Datasets are available through the corresponding author upon reasonable request.
